# Why robust identification of rare yeasts is the need of the hour

**DOI:** 10.1128/spectrum.01419-25

**Published:** 2025-12-17

**Authors:** Anuradha Marathe, Brittany O'Brien, YanChun Zhu, Mayuri Vaidya, Sudha Chaturvedi

**Affiliations:** 1 New York State Department of Health, Wadsworth Center Mycology Laboratory 1094, Albany, New York, USA; 2 Department of Biomedical Sciences, College of Integrated Health Sciences, University at Albany 1084, Albany, New York, USA; Debreceni Egyetem, Debrecen, Hungary

**Keywords:** rare yeasts, MALDI, public health

## Abstract

Infections caused by rare yeasts are surging, mainly due to the increase in immunocompromised or seriously ill patients. With the increasing clinical significance of rare yeasts, there is an urgent need for a rapid diagnostic method. The current process relies on the PCR of the ribosomal gene’s internal transcribed spacer (ITS) region, followed by sequencing and BLAST search, which are labor- and time-intensive. Matrix-assisted laser desorption ionization-time of flight mass spectrometry (MALDI-TOF MS) has revolutionized the rapid identification of yeasts at the species level. One limitation of MALDI-TOF MS is the inadequacy or lack of main spectrum profiles (MSPs) in the reference library, which results in low scores and no organism identification. The focus of this study was to create an in-house Bruker library for 141 rare yeast isolates, representing 25 genera and 68 species of which 31 species had no MSPs, and 37 species were poorly represented in the commercial Bruker library. Protein extraction using the ethanol/formic acid method with a few yeasts requiring a bead-beating step before protein extraction was employed for library construction. A minimum of 20 spectra and a log (score) of ≥1.9 were used for species-level identification. Upon library construction, all 141 rare yeast isolates were successfully identified to the species level with a log (score) of 2.0 or higher. In conclusion, MALDI-TOF MS with enriched in-house Bruker library is a powerful tool for identifying rare yeasts, offering speed, accuracy, and clinical relevance.

**IMPORTANCE:**

The infection caused by rare yeasts among immunocompromised or seriously ill patients is on the rise. Therefore, a comprehensive and rapid method for accurate identification is urgently needed. We have addressed this need by enriching the in-house Bruker library by adding 141 rare yeast isolates, representing 25 genera and 68 species. This comprehensive update has resulted in improved turnaround time, a development that will significantly enhance patient management by providing more timely and accurate identification of rare yeast infections, thereby improving patient outcomes. Moreover, the enriched library spectrum profiles generated in this study have been made publicly available through the Centers for Disease Control and Prevention’s MicrobeNet database (https://MicrobeNet.cdc.gov), enabling collaboration in the scientific community.

## INTRODUCTION

Fungal pathogens are responsible for at least 13 million infections and 1.5 million deaths globally per year, primarily in the immunosuppressed or those hospitalized with serious, underlying diseases (1). Over the last three decades, the incidence of hospital-acquired fungal infections has increased. A single fungal pathogen can infect multiple tissues in an immunocompromised patient and can undergo morphogenic shifts during infection (2). As a result, symptoms of an invasive fungal infection are frequently nonspecific, and early diagnosis is often challenging to establish. Generic therapy carries risks of both treatment-associated toxicity and development of resistance in the pathogens (3). The most common fungal disease in hospitalized patients is invasive candidiasis, which is associated with mortality exceeding 40%, even with treatment (3). The spectrum of pathogens causing invasive candidiasis has also shifted from *Candida albicans* to non-*albicans Candida* species. There is also a rise in rare yeasts belonging to *Candida* or non-*Candida* genera as emerging threats in the healthcare setting due to their high virulence, proliferation within the body, and their drug-resistance profile (4–6). Therefore, the role of diagnostic mycology laboratories in adapting to the emergence of the new and rare yeasts is more crucial than ever (7).

Existing commercial identification systems, which traditionally identify pathogenic yeasts based on morphological and biochemical characteristics, are time-consuming, have limited databases, and are designed to identify only the more common medically important yeasts (8). On the other hand, the polymerase chain reaction (PCR) and sequencing of the ribosomal genes, the internal transcribed spacer (ITS) and D1/D2, are the gold standard methods for yeast identification (8, 9). These methods are excellent but time-consuming, which can lead to delays in patient care.

Around 2010, the introduction of matrix-assisted laser desorption ionization-time of flight mass spectrometry (MALDI-TOF MS) technology into the clinical microbiology field was a game-changer, as clinical laboratories could identify organisms from crude protein suspensions within minutes, at very low processing costs, and minimal processing time. Over the recent years, MALDI-TOF MS has been successfully used to identify numerous yeast species (9–13). One limitation of MALDI-TOF MS is the absence or inadequacy of main spectrum profiles (MSPs) in the reference library, which can lead to low scores and no organism identification (14, 15). Moreover, cases of infection by newly emerging and newly pathogenic yeasts, including *Candida auris,* are on the rise (16–20). Another concern is the increasing incidences of rare yeast infections, such as *Candida blankii* and *Debaryomyces hansenii* in immunocompetent individuals over the last decade (21, 22). Under these circumstances, improving the MALDI database and having a better representation of the rare and emerging yeasts is the need of the hour and a key factor for the accurate identification of rare yeasts. For instance, in 2016, with the rapid emergence of *C. auris* as a global threat, many laboratories developed their in-house supplemental databases, which helped them accurately identify *C. auris* (23–26). This demonstrates that in-house developed databases can significantly improve performance and serve as a valuable measure for rapid response to emerging pathogens, provided they are rigorously validated and interpreted with care (10). In this investigation, we describe the utility of our in-house developed Bruker MALDI-TOF MS library as a powerful tool for identifying rare yeasts, offering speed, accuracy, and clinical relevance.

## MATERIALS AND METHODS

**Fig 1:**
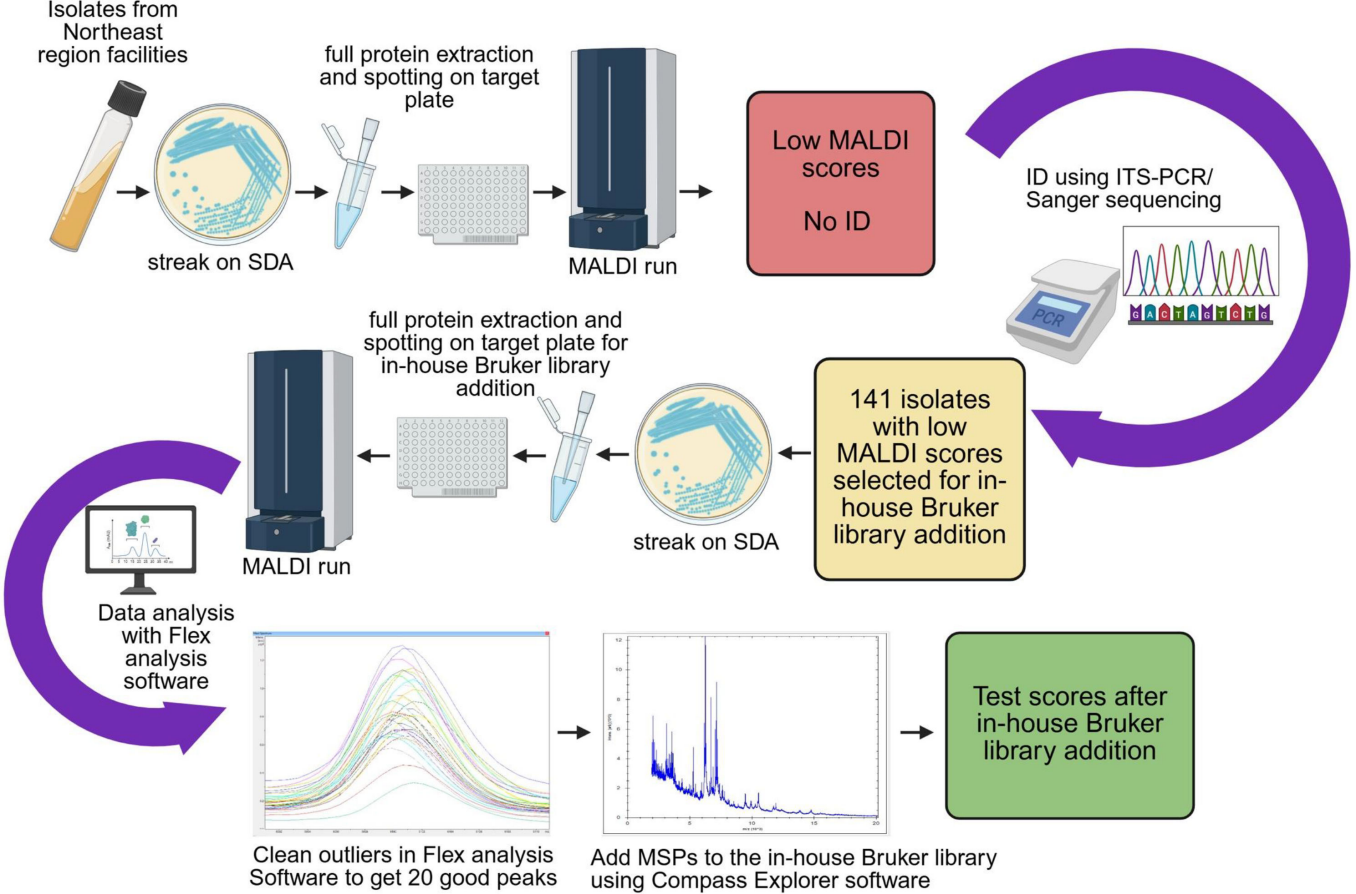
Flowchart of library addition of rare yeasts. A flowchart illustrating the entire process of creation of an in-house Bruker library of rare yeasts. The schematic was created with BioRender.com. SDA, Sabouraud dextrose agar.

For this study, 141 rare yeast isolates representing 68 species and 25 genera, identified by sequencing of the ITS region of the ribosomal gene, were selected. The ITS sequences of these rare yeasts were submitted to GenBank with accession numbers PQ644596–PQ644604, PQ644607–PQ644612, PV029103–PV029223, and PV029329. The majority of these isolates could not be identified by MALDI-TOF MS and were added to the library, while few were added for the purpose of library enrichment. All isolates in this investigation were obtained from the Mycology Culture Collection Repository (MCCR) (https://www.wadsworth.org/programs/id/mycology/culture-collection-repository). For MALDI-TOF MS protein extraction, yeasts were grown on Sabouraud dextrose agar (SDA) plates overnight at 30°C. The protein from each yeast isolate was extracted using ethanol/formic acid extraction method (Bruker Daltonics, Germany) (27). In brief, yeast colonies were placed into a 1.5-mL centrifuge tube containing 300 µL of liquid chromatography mass spectrometry (LCMS) grade water and mixed thoroughly, followed by addition of 900 µL of absolute ethanol. The tubes were centrifuged at 13,200 RPM for 2 min at room temperature (RT), the supernatant was discarded, and the pellet was air-dried for 30 min. The air-dried pellet was then mixed with 25 µL of 70% formic acid, incubated at RT for 10 min, followed by addition of 25 µL of acetonitrile, mixing thoroughly, and centrifuging at 13,200 RPM for 2 min at RT. One microliter of the supernatant was spotted on 12 spots of a 96-spot target plate (Bruker Daltonics, Germany). One microliter of Bacterial Test Standard (BTS, Bruker Daltonics, Germany) was spotted on one spot of the plate as a positive control. All spots were air-dried for approximately 5 min, and then each spot was overlaid with 1 µL of α-cyano-4-hydroxycinnamic acid (HCCA) matrix and air-dried completely (~10–15 min) before the MALDI-TOF MS run. Six isolates belonging to five rare yeast species, which failed to yield protein spectra in MALDI, were mixed with 25 µL of 70% formic acid and subjected to bead-beating at 4,700 RPM for 45 s (Precellys24 Homogenizer) and incubated at RT for 10 min followed by protein extraction and supernatant spotting on target plate as described above. MSPs were obtained following the manufacturer’s guidelines by using the MALDI Biotyper software version 3.1 (Bruker Daltonics, Germany). All spectra were scrutinized using the Flex analysis software (Bruker Daltonics, Germany). Spectra with outlier, low-quality, and noisy peaks were deleted, and a minimum of 20 high-quality spectra were selected for each yeast isolate. These spectra were stored in the in-house Bruker library as a reference MSP using Compass Explorer (Bruker Daltonics, Germany). According to Bruker, the resemblance between an unknown specimen spectrum and reference spectra is indicated by a log (score), which will be henceforth referred to as “MALDI score” (28). A MALDI score of ≥1.9 was considered as the cutoff for species-level identification. The entire process of library addition of rare yeasts has been shown in a flowchart (Fig. 1).

Statistical analysis of the MALDI data were performed using the built-in analysis feature of Graphpad Prism software (version 9.5.1). The paired *t*-test was used for comparison of two groups (MALDI scores before addition and MALDI scores after addition). Results were considered significant at a *P*-value < 0.05.

To determine genotypic differences within the yeast species, ITS sequences were aligned using the Create Alignment function in the QIAGEN CLC Genomics Workbench (version 24.0.2). The neighbor-joining algorithm and Jukes-Cantor nucleotide substitution model were implemented for constructing a phylogenetic tree representing all rare yeasts, as well as several individual phylogenetic trees in the QIAGEN CLC Genomics Workbench (version 24.0.2). All the gaps were excluded from the analysis, and branch support was ascertained using 1,000 bootstrap replicates.

## RESULTS AND DISCUSSION

**Fig 2:**
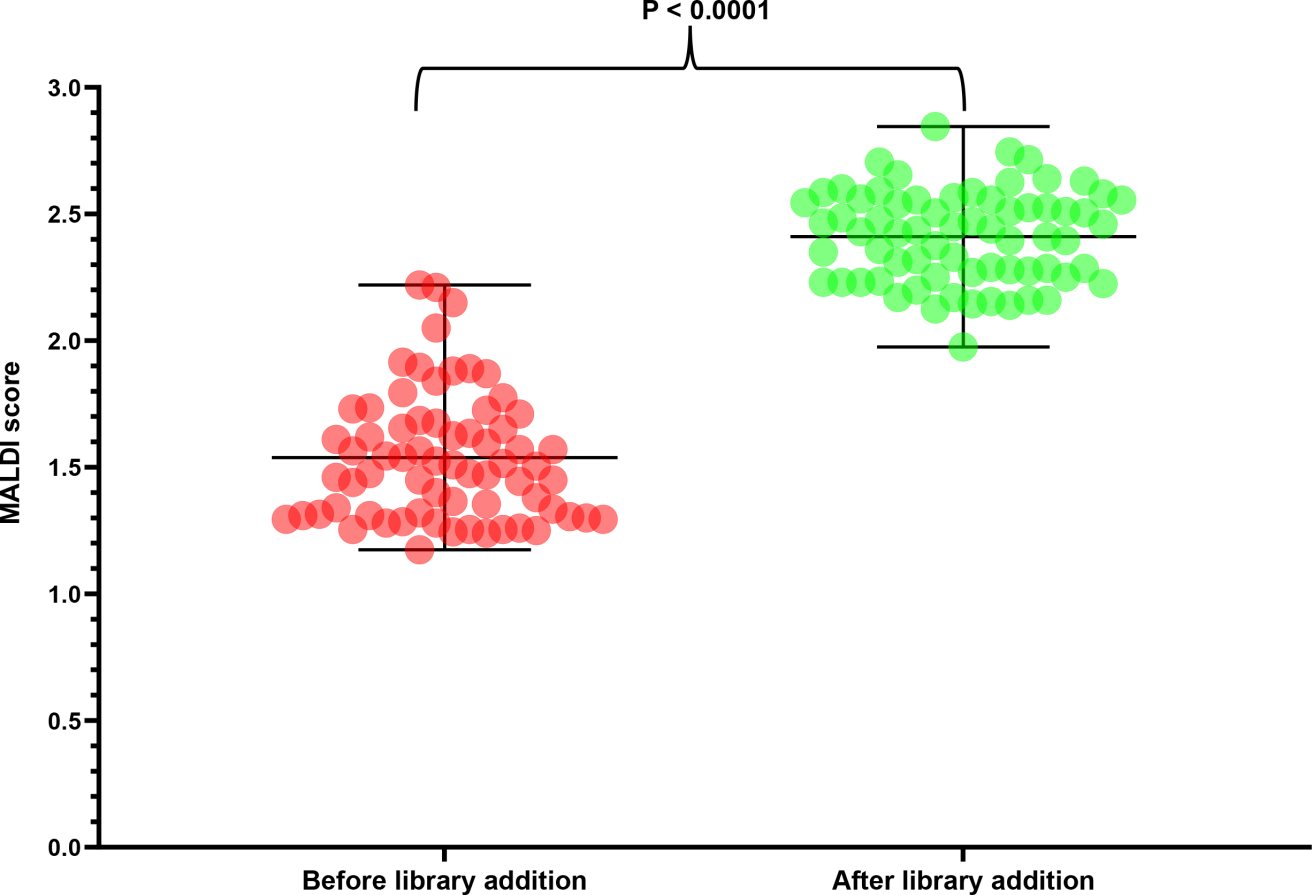
Distribution of MALDI scores of rare yeasts before and after library addition. A column graph was created in GraphPad Prism version 9.5.1 using the scatter plot function, which shows mean and ranges as dots. Red dots represent the mean MALDI score before, and green dots represent the mean MALDI scores after the addition of rare yeasts into the in-house Bruker library.

We successfully added 141 yeast isolates representing 68 species and 25 genera to the in-house Bruker library. The library addition resulted in accurate identification of rare yeasts to the species level with a MALDI score of 2.0 or above. The ethanol/formic acid protein extraction was adequate for 135 isolates (95.7%), representing 63 rare yeasts for identification, whereas six isolates (4.3%) representing five species (*Candida fermenticarens*, *Candida sake*, *Candida terebra*, *Candida lactosa*, *Trichomonascus ciferrii*) required an additional bead-beating step (Tables 1 and 2). The in-house Bruker library was further evaluated by testing 47 rare yeast isolates in a blinded fashion, and MALDI score of 2.0 or above confirmed the accuracy of the in-house Bruker library (Table S3). In summary, addition of rare yeasts to the in-house Bruker library led to a significant (*P* < 0.0001) improvement in MALDI score distribution (Fig. 2).

**TABLE 1:** A list of rare yeasts with no MSPs in the Bruker library^*a*^

Yeast species (old name)	Yeast species (new name)	# of isolates added to in-house Bruker library	MALDI score before library enrichment	MALDI score after library enrichment	Known to cause human infections
*Candida diddensiae*	–	2	1.26/1.25	2.53/2.58	Invasive (29)
*Candida etchellsii*	*Starmerella etchellsii*	1	1.20/1.28	2.29/2.03	Unknown
*Candida ethanolica*	–	1	1.23/1.29	2.54/2.58	Unknown
*Candida fermentati*	*Meyerozyma caribbica*	7	1.59/1.63	2.24/2.34	Invasive (30)
*Candida fermenticarens^a^*	–	1	1.29/1.30	2.41/2.52	Unknown
*Candida lactativora*	–	1	1.24/1.35	2.46/2.36	Unknown
*Candida lactosa^a^*	–	1	1.23/1.39	2.47/2.40	Unknown
*Candida melibiosica*	–	1	1.30/1.33	2.67/2.64	Unknown
*Candida naganishii*	*Debaryomyces nepalensis*	1	1.22/1.29	2.51/2.50	Unknown
*Candida phangngaensis*	–	2	1.29/1.31	2.51/.260	Unknown
*Candida quercitrusa*	–	1	1.54/1.60	2.17/2.14	Invasive (17)
*Candida railenensis*	–	1	1.23/1.27	2.23/2.23	Unknown
*Candida sake^a^*	–	1	1.39/1.32	2.50/2.61	Invasive (31)
*Candida sorbosivorans*	*Starmerella sorbosivorans*	3	1.41/1.67	2.53/2.55	Unknown
*Candida steatolytica*	–	4	1.66/1.65	2.52/2.50	Unknown
*Candida stellimalicola*	–	3	1.28/1.28	2.50/2.55	Invasive (18)
*Candida subhashii*	–	1	1.14/1.21	2.23/2.27	Invasive (32)
*Candida terebra^a^*	–	1	1.22/1.35	2.72/2.77	Unknown
*Candida thasaenensis*	–	3	1.50/1.30	2.20/2.26	Unknown
*Cryptococcus flavus*	*Saitozyma flava*	1	1.28/1.23	2.20/2.20	Unknown
*Cryptococcus wisconsinensis*	*Papiliotrema wisconsinensis*	1	1.49/1.45	2.31/2.33	Unknown
*Pichia farinosa*	–	1	1.54/1.62	2.20/2.35	Invasive (33)
*Rhodotorula laryngis*	*Cystobasidium laryngis*	1	1.37/1.36	2.27/2.05	Unknown
*Rhodotorula slooffiae*	*Cystobasidium slooffiae*	9	1.58/1.55	2.18/2.16	Unknown
*Torulaspora globosa*	–	1	1.26/1.23	2.40/1.85	Unknown
*Trichosporon dermatis*	*Cutaneotrichosporon dermatis*	2	1.37/1.76	2.40/2.39	Non-invasive (34)
*Trichosporon domesticum*	–	1	1.77/1.65	2.57/2.33	Unknown
*Trichosporon insectorum*	–	3	1.51/1.51	2.51/2.50	Invasive (35)
*Trichosporon montevideense*	–	1	1.62/1.63	2.83/2.86	Invasive (36)
*Vishniacozyma foliicola*	–	1	1.46/1.22	2.62/2.64	Unknown
*Yarrowia divulgata*	–	1	1.47/1.42	2.51/2.34	Unknown

^*a*^ Additional step of bead-beating required for protein extraction followed by library addition; “–" indicates no change in the current name.

**TABLE 2:** A list of rare yeasts with multiple MSPs in Bruker library with low MALDI score^*a*^

Yeast species (old name)	Yeast species (new name)	# of isolates in Bruker library	# of isolates added to in-house Bruker library	MALDI score before library enrichment	MALDI score after library enrichment	Known to cause human infections
*Apiotrichum mycotoxinivorans*	*–*	2	1	2.07/2.23	2.34/2.36	Invasive (37)
*Candida allociferrii*	*–*	2	1	1.66/1.64	2.54/2.63	Invasive (38)
*Candida blankii*	*–*	4	13	1.51/1.54	2.29/2.28	Invasive (39)
*Candida bracarensis*	*Nakaseomyces bracarensis*	3	2	1.81/1.78	2.29/2.27	Invasive (6)
*Candida catenulata*	*Diutina catenulata*	7	2	1.66/1.71	2.29/2.25	Invasive (40)
*Candida ciferrii^a^*	*Trichomonoascus ciferrii*	3	2	1.51/1.52	2.47/2.45	Invasive (41)
*Candida cylindracea*	*–*	1	2	1.48/1.40	2.55/2.47	Unknown
*Candida fabianii*	*Cyberlindnera fabianii*	8	3	1.89/1.87	2.28/2.22	Invasive (42)
*Candida famata*	*Debaryomyces hansenii*	11	8	1.54/1.60	2.49/2.46	Invasive (6)
*Candida galli*	*–*	1	2	1.46/1.46	2.58/2.60	Invasive (43)
*Candida intermedia*	*–*	9	2	1.70/1.76	2.47/2.15	Invasive (6)
*Candida kefyr*	*Kluyveromyces marxianus*	17	2	1.89/1.89	2.31/2.35	Invasive (6)
*Candida metapsilosis*	*–*	11	7	1.75/1.80	2.49/2.48	Invasive (44)
*Candida nivariensis*	*Nakaseomyces nivariensis*	8	2	1.47/1.48	2.37/2.51	Invasive (6)
*Candida norvegensis*	*Pichia norvegensis*	8	1	1.48/1.47	2.05/1.90	Invasive (6)
*Candida oleophila*	*–*	1	1	1.39/1.25	2.57/2.63	Unknown
*Candida pararugosa*	*Wickerhamiella pararugosa*	2	3	2.23/2.21	2.57/2.48	Invasive (16)
*Candida pelliculosa*	*Wickerhamomyces anomalus*	10	3	1.75/1.70	2.41/2.45	Invasive (6)
*Candida rugosa*	*Diutina rugosa*	5	3	1.52/1.49	2.17/2.12	Invasive(6)
*Candida sojae*	*–*	3	2	1.73/1.74	2.37/2.38	Invasive(45)
*Candida lusitaniae*	*Clavispora lusitaniae*	15	3	1.65/1.62	2.59/2.50	Invasive (6)
*Cryptococcus albidosimilis*	*–*	2	4	1.42/1.48	2.28/2.18	Invasive (46)
*Cryptococcus albidus*	*Naganishia albida*	1	3	1.34/1.42	2.18/2.10	Invasive (47)
*Cryptococcus diffluens*	*Naganishia diffluens*	3	4	1.64/1.55	2.32/2.25	Invasive (48)
*Cryptococcus flavescens*	*–*	2	1	1.40/1.69	2.25/2.20	Invasive (19)
*Cryptococcus gattii* VGI	*–*	15	1	1.56/1.68	2.30/2.64	Invasive (49)
*Cryptococcus liquefaciens*	*Naganishia liquefaciens*	1	1	1.37/1.25	2.62/2.54	Invasive (50)
*Exophiala dermatitidis*	*–*	3	8	1.87/1.81	2.38/2.34	Invasive (51)
*Hanseniaspora uvarum*	*–*	13	1	1.88/1.86	2.32/2.47	Unknown
*Pichia terricola*	*–*	3	1	1.98/1.81	2.42/2.05	Invasive (52)
*Trichosporon asahii*	*–*	10	1	1.26/1.30	2.62/2.51	Invasive (36)
*Trichosporon coremiiforme*	*-*	2	1	2.17/2.25	2.53/2.72	Invasive (36)
*Trichosporon debeurmannianum*	*Cutaneotrichosporon debeurmannianum*	2	1	1.23/1.38	2.63/2.54	Non-invasive (20)
*Trichosporon faecale*	*–*	1	2	1.46/1.44	2.61/2.67	Invasive (36)
*Trichosporon ovoides*	*–*	4	1	1.67/1.68	2.69/2.72	Invasive (34)
*Trichosporon terricola*	*Cutaneotrichosporon terricola*	2	1	1.32/1.35	2.74/2.69	Unknown
*Zygosaccharomyces bailii*	*–*	3	1	2.01/2.09	2.16/2.18	Unknown

^*a*^ Additional step of bead-beating required for protein extraction followed by library addition; “–" indicates no change in the current name.

**Fig 3:**
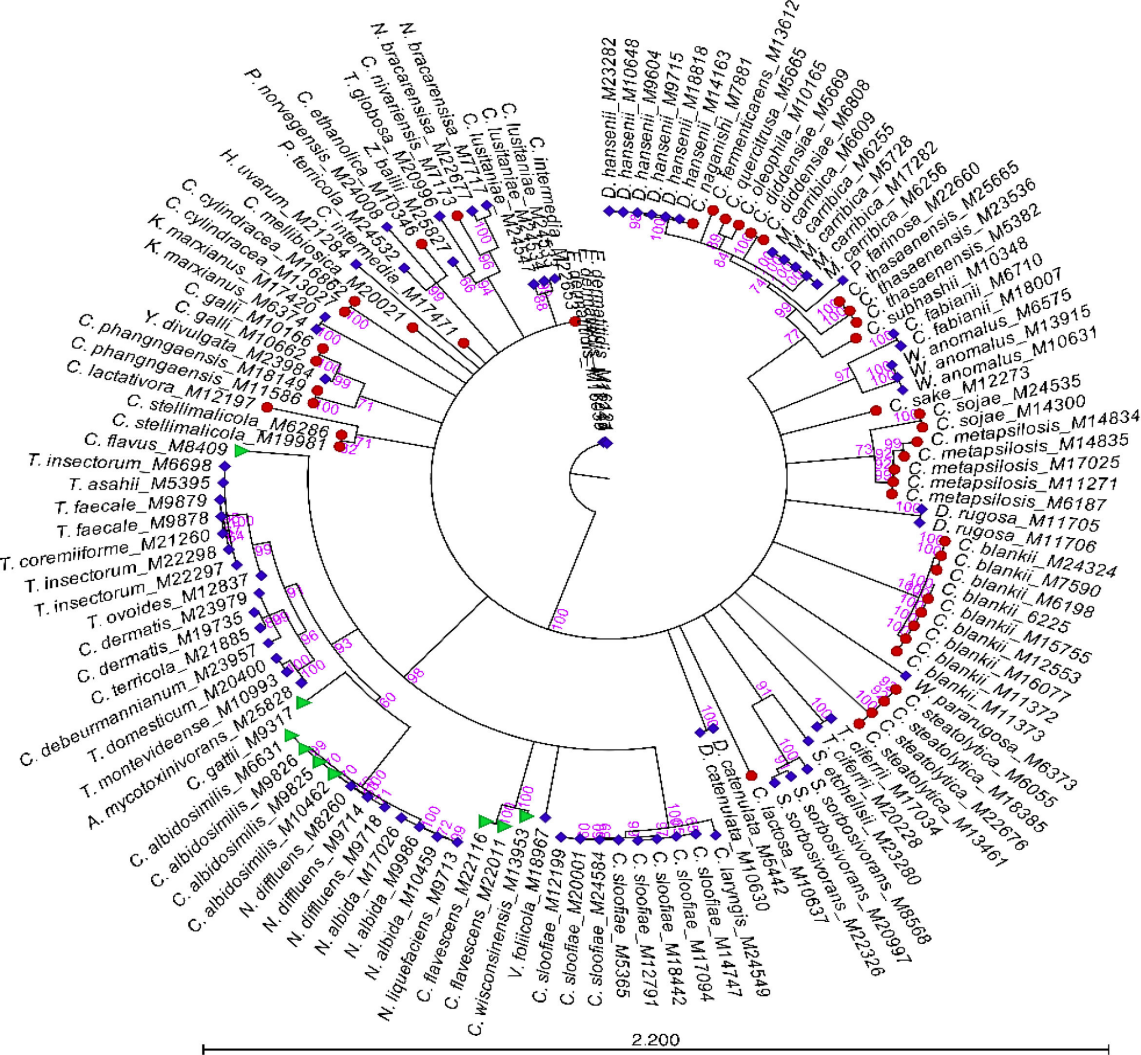
Phylogenetic analysis of rare yeasts. The phylogenetic tree of 132 isolates of 65 rare yeast species was constructed using the QIAGEN CLC Genomics Workbench (version 24.0.2). Multiple alignment of *ITS* genes was performed using the Create Alignment function in the CLC Genomics Workbench. The neighbor-joining algorithm and Jukes-Cantor nucleotide substitution model were implemented. All gaps were excluded from the analysis, and branch support was ascertained using 1,000 bootstrap replicates. The numbers next to the branches indicate the bootstrap values.

**Fig 4:**
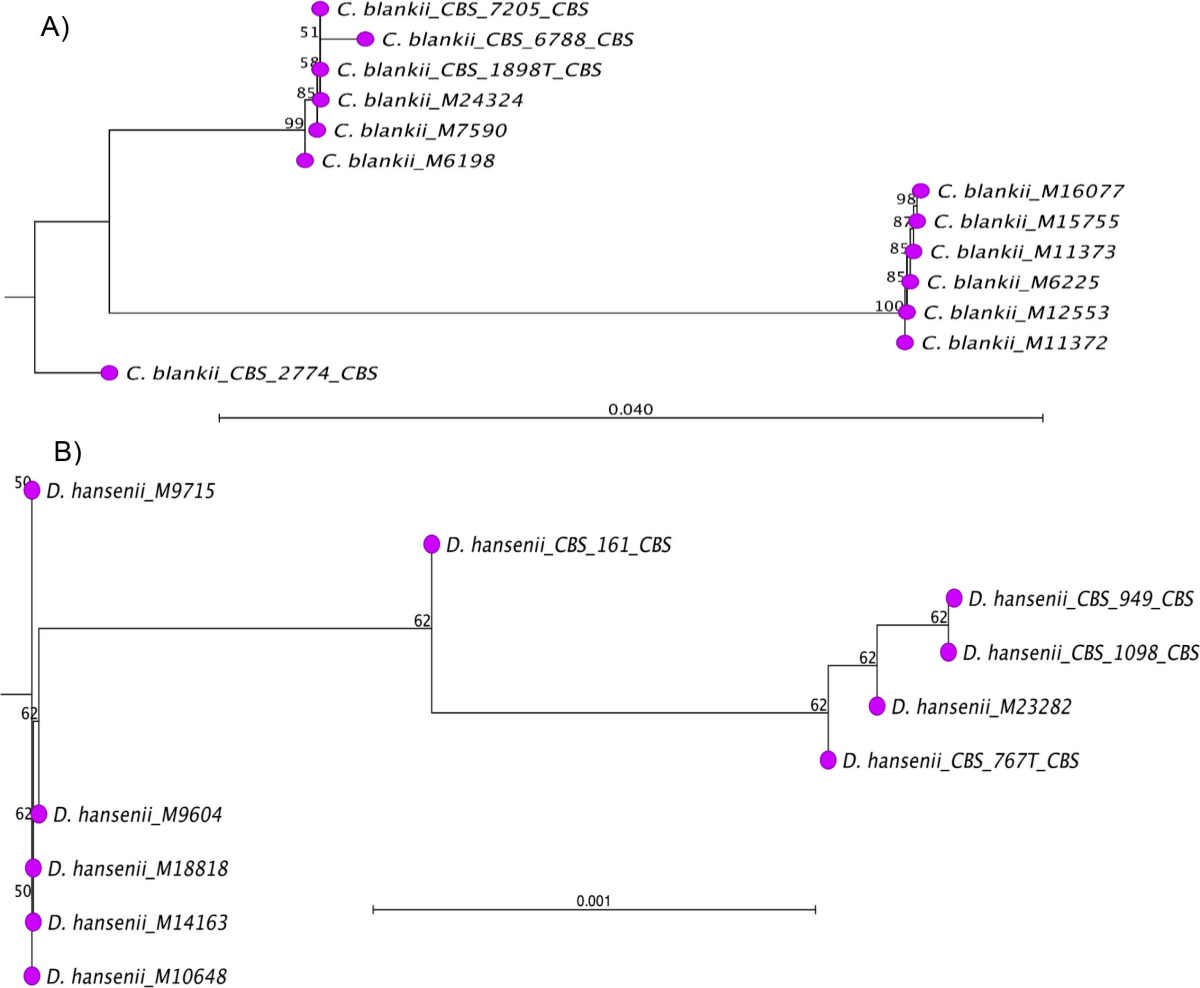
Phylogenetic analysis of *Candida blankii* and *Debaromyces hansenii* based on geographical distribution. Multiple alignment of *ITS* genes was performed using the Create Alignment function of the QIAGEN CLC Genomics Workbench (version 24.0.2). The neighbor-joining algorithm and Jukes-Cantor nucleotide substitution model were implemented. All gaps were excluded from the analysis, and branch support was ascertained using 1,000 bootstrap replicates. The numbers next to the branches indicate the bootstrap values. Phylogenetic analyses of *C. blankii* (**A**) and *D. hansenii* (**B**) are shown.

Of the 68 species of 25 genera of rare yeasts added, 31 species of 11 genera had no reference spectra in the commercial Bruker library. Therefore, the lack of identification for these yeasts by MALDI-TOF MS was not surprising (Table 1). However, despite some of the rare yeasts being adequately represented in the commercial Bruker library, they failed to yield a good score for species-level identification (Table 2). The multiple alignments of ITS sequences revealed single-nucleotide polymorphisms (SNPs) in isolates within a species. Some of these species were *C. blankii, Candida cylindracea, Candida diddensiae, Candida stellimalicola, Cystobasidium sloofiae, Naganishia albida, Starmerella sorbosivorans,* and *Cutaneotrichosporon dermatis,* resulting in clustering on different branches of the phylogenetic tree (Fig. 3). These results indicate that there are several genotypes within the species, and library enrichment with all possible genotypes is key for successful identification through MALDI-TOF MS. When ITS sequences of Bruker *C. blankii* and *D. hansenii* (formerly known as *Candida famata*) isolates were compared with ITS sequences of *C. blankii* and *D. hansenii* isolates from the northeastern United States, a distinct clustering of Bruker isolates to that of U.S. isolates was observed (Fig. 4A and B). These results indicate the possible cause for low MALDI score in the initial phase of this investigation, and adding local strains to the library yielded successful identification (Table 2). The Bruker isolates were derived from other parts of the globe (Table S1), whereas the local isolates were derived from the northeastern United States (Table S2). Genetic variation in isolates can be influenced by geographical distribution and other factors such as climate, ecological niches, changes in chromosomal copy number, and hyphal network. According to a haplotype and network analysis, high intraspecific variability of ITS sequences was observed in pathogenic yeast species, including *Candida albicans*, *Candida tropicalis*, *Nakaseomyces glabratus* (formerly known as *Candida glabrata*), and *Clavispora lusitaniae* (53). Research suggests that species with high genetic diversity are most frequently human commensals, and this finding could explain the existence of additional genetic adaptation within normal microbiota with older evolutionary origins (23, 24).

The antifungal susceptibility testing (AFST) data available for certain rare yeasts were analyzed retrospectively (Table S4). The resistance pattern of rare yeasts varied from low to high MIC for azoles, echinocandins, amphotericin B, and 5-fluorocytosine (Table S4). Although no breakpoints are available for rare yeasts, several of them, including *Candida allociferrii*, *C. blankii, Candida oleophila, Candida subhashii, Kluyveromyces marxianus*, *Nakaseomyces bracarensis*, and *Wickerhamiella pararugosa,* showed high MIC against first-line drug fluconazole (MIC range 16 to >256 µg/mL), *Apiotrichum myxotoxinivorans*, *D. hansenii*, and *Trichosporon asahii* showed high MIC against amphotericin B (MIC range 16 to >32 µg/mL), and *Apiotrichum myxotoxinivorans*, *C. blankii*, *Candida lactativora*, *Candida steatolytica*, *Cutaneotrichosporon* spp., and *Naganishia* spp. showed high MIC against echinocandins (MIC range 4 to >16 µg/mL) (Table S4).

Additionally, we also calculated MIC_50_ and MIC_90_ for yeast species with 10 or more isolates, and the majority of these species showed elevated MIC against fluconazole and few against voriconazole or amphotericin B, the drugs often used as the first choice of antifungals in the empirical antifungal therapy (Table S5). These results are not surprising as several of these rare yeasts have shown high MIC against azoles, echinocandins, and amphotericin B (6, 7, 29, 30). Of particular interest, *C. blankii*, which was believed to be non-pathogenic to humans until 2015, was reported as the cause of infection in 12 cases worldwide, and nine of them came from hospital-associated outbreaks in India (21, 54). In another case of *C. blankii* bloodstream infection in New York, the second *C. blankii* case in the United States, an unidentified yeast and *Aspergillus niger* were isolated from respiratory secretions. Voriconazole was administered as an empiric drug for both *A. niger* and the unidentified yeast. Unfortunately, the patient died within a week of isolating the yeast. Eventually, *C. blankii* was identified as the causative agent of fungemia in our laboratory by ITS-PCR and sequencing, and AFST revealed azole resistance (39). Such cases reinforce the significance of rapid and accurate identification of rare yeasts.

While commercial databases, such as Bruker or Vitek MS undergo frequent updates, there is a need for in-house library enrichment to include isolates of adequate worldwide representation. In the present investigation, MALDI library enrichment reduced the turnaround time of rare yeasts identification without the need to perform ITS-PCR and sequencing, which is time-consuming and labor-intensive. Taverna et al. developed an in-house MALDI database by adding regional strains and demonstrating improved MALDI scores (13). Similarly, studies all over the world have shown successful addition of regional isolates of *Malassezia* spp., *C. auris*, *Candida haemulonii*, *Candida duobushaemulonii*, *Candida krusei*, and *Kodamaea ohmeri* to their in-house MALDI databases (23, 24, 55). As a public health laboratory of New York State and the Centers for Disease Control and Prevention’s Northeast Regional Laboratory for the Antibiotic Resistance Laboratory Network of the United States, we urgently need to have robust identification technologies, including MALDI-TOF MS. Implementing an in-house Bruker library will enable the correct identification of an increasing number of rare yeasts and help in administering proper treatment options while controlling spread to the vulnerable population. The enriched library spectrum profiles generated in this study, comprising local isolates of rare yeasts from the northeastern United States, are now available publicly through the Centers for Disease Control and Prevention’s MicrobeNet database (https://microbenet.cdc.gov/).

## Data Availability

The ITS sequences of the rare yeasts were submitted to GenBank with accession numbers PQ644596–PQ644604, PQ644607–PQ644612, PV029103–PV029223, and PV029329. The enriched library spectrum profiles generated in this study are available publicly through the Centers for Disease Control and Prevention’s MicrobeNet database (https://microbenet.cdc.gov/).

## SUPPLEMENTAL MATERIAL

Supplemental tables spectrum.01419-25-s0001.xlsx Tables S1 to S5.

ASM does not own the copyrights to Supplemental Material that may be linked to, or accessed through, an article. The authors have granted ASM a non-exclusive, world-wide license to publish the Supplemental Material files. Please contact the corresponding author directly for reuse.
